# Selective Co(II) and Ni(II) Separation Using the Trihexyl(tetradecyl)phosphonium Decanoate Ionic Liquid

**DOI:** 10.3390/molecules29194545

**Published:** 2024-09-25

**Authors:** Anđela Kovačević, José Alejandro Ricardo García, Marilena Tolazzi, Andrea Melchior, Martina Sanadar

**Affiliations:** 1 Chemical Technologies Laboratories, Polytechnic Department of Engineering, University of Udine, Via Cotonificio 108, 33100 Udine, Italy; kovacevic.andela@spes.uniud.it (A.K.); ricardogarcia.josealejandro@spes.uniud.it (J.A.R.G.); marilena.tolazzi@uniud.it (M.T.); 2 Centre de Biophysique Moléculaire, CNRS, UPR 4301, Université d’Orléans, Rue Charles Sadron, 45071 Orléans, Cedex 2, France; martina.sanadar@cnrs-orleans.fr

**Keywords:** ionic liquids, cobalt, nickel, separation, polymer inclusion membranes

## Abstract

The room temperature ionic liquid trihexyl(tetradecyl)phosphonium decanoate ([P_66614_][Dec]) was employed in the liquid-liquid extraction of Co(II) from hydrochloric acid solutions in the presence of Ni(II). The extraction performance in liquid-liquid separations showed a strong dependence on the acid content of the feed aqueous solution. The best performance in terms of extracted cobalt and selectivity was obtained when the feed contained a HCl concentration above 6 M On the contrary, when the experiment was performed in absence of HCl, a lower extraction and Co/Ni selectivity were obtained. This behavior has been rationalized by considering the protonation of the [Dec]^−^ anion and the different Co(II)/Ni(II) speciation in HCl media. Moreover, polymer inclusion membranes (PIMs) were prepared using PVC and [P_66614_][Dec] at different weight rations. Only the PIM formulated with a 30:70/PVC:[P_66614_][Dec] weight ratio demonstrated effective extraction of Co(II) from the HCl solution. The extraction efficiency and selectivity of the PIM was comparable to that from biphasic liquid experiments at 8 M HCl. The results of this study constitute a promising background for further practical developments of carboxylate-based ILs applied in Co/Ni separations.

## 1. Introduction

It is estimated that more than 1.2 million tons of Li-ion batteries enter the European Union each year, with global demand predicted to grow considerably over the next five years [1]. As a result, the demand for Co(II), an essential element for manufacturing several types of Li-ion battery cathodes, is projected to increase 20 times by 2050 [2,3]. Over half of the global Co supply comes from the Democratic Republic of the Congo, where extraction is associated with significant social and political issues [4]. This steep increase in consumption is in turn projected to lead to corresponding waste generation, which needs to be addressed to protect the environment and to recover valuable critical raw materials (CRMs) [5,6].

Several types of extractive metallurgy still face a difficult challenge in separating Co(II) from Ni(II) due to the similarities in the chemical properties of these two elements [7]. The Co/Ni separation holds crucial importance for the production of these transition metals and their corresponding salts from primary ores, including Ni-Cu sulfides [8] and Ni laterite ores [9]. End-of-life battery recycling is also an area of active research because of the high material value and potential toxicity of the waste [10]. Recycling Co from spent Li-ion battery cathodes, such as nickel manganese cobalt oxide (NMC), could significantly reduce the pressure due to mining activity.

Hydrometallurgical processes [11] have the notable advantages of producing highly pure products and being much less energy intensive than pyrometallurgical processes [12]. However, the volatile organic compounds (VOCs) employed as solvents in combination with extracting ligands pose significant safety and environmental risks. Acidic extractants like organophosphorus acids (D2EHPA, PC88A, Cyanex 272, and Cyanex 302) [13,14] have been used for Co-Ni separations but exhibit low selectivity and require strict pH control [15,16,17]. Moreover, combined hydro/pyrometallurgical approaches have also been considered [18].

More recently, ionic liquids (ILs) have been proposed as a safer and more efficient media for metal ion extraction and selective separation [11,19,20]. Fluorinated hydrophobic ILs have been employed for solvent extraction [21,22,23,24,25,26]; however, these ILs do have certain disadvantages, including their high cost and persistence in the environment [27]. Such drawbacks could be reduced if the components of the ILs are derived from renewable biomaterials [28], thus being less expensive and more sustainable. In long-chain fatty acid ionic liquids (LCFA-ILs) the main physicochemical properties are strongly related to the alkyl chain length and the degree of saturation [29,30]. These ILs have been explored as green alternatives to conventional hydrophobic ILs in liquid–liquid extraction, where they have shown the ability to effectively extract metals [31,32] and phenols [33] from aqueous solutions. Furthermore, research has also indicated that these ILs possess antimicrobial properties [34].

Many applications of phosphonium-based ILs in metal separations have been reported in the literature. Mo(VI) with trihexyl(tetradecyl)phosphonium bromide, [P_66614_][Br] [35], Pd(II) extraction as well as Fe(III) separation from Ni(II) with trihexyl(tetradecyl)phosphonium chloride, [P_66614_][Cl] [36,37], extraction of Eu(III) and other rare-earth elements with trihexyl(tetradecyl)phosphonium nitrate, [P_66614_][NO_3_] [38], and Co(II) from Sm(III) using [P_66614_][Cl] [39]. As far as the application of phosphonium-based ILs in Li-ion cathode battery recycling is concerned, several works have been published [40,41,42].

Besides the simple liquid-liquid separations, polymeric membranes can be employed in combination with ILs to fabricate composite systems (polymer inclusion membranes, PIMs). Among various membrane technologies, PIMs stand out as self-supported liquid membranes, gaining prominence due to their straightforward preparation, reusability [43,44], stability [44,45], and low toxicity [46,47,48]. The PIM is placed between the feed aqueous phase containing the metals and the receiving phase where the separated metals are stripped [49]. One notable advantage of PIMs is the reduced amount of IL employed with respect to liquid-liquid biphasic systems, which is important, as one of the main issues limiting industrial applications of ILs is their high cost. Moreover, in the membrane-based process, the extraction and stripping occur in a single stage.

**Figure 1:**
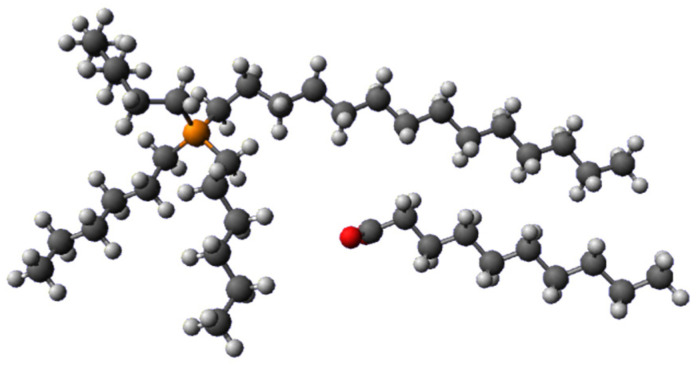
Structure of [P_66614_][Dec].

In this framework, the aim of the present study is to assess the application of [P_66614_][Dec] (trihexyl(tetradecyl)phosphonium decanoate, Figure 1) in the extraction of Co(II) from an aqueous phase and the separation from Ni(II). The carboxylate moiety can act as a complexing group and therefore allow extractions without the use of auxiliary ligands in the organic phase. Moreover, the decanoate anion can be considered as a model of a biomass-derived fatty acid which is more biocompatible than other anions employed in commercial hydrophobic ILs [28].

While this IL has been previously mainly employed in the extraction of organic molecules from aqueous solutions [50,51,52,53], to date, only one study on metal ion extraction (La(III) and Yb(III) [54]) has been published.

In the present study, first the performance of the IL is studied in liquid-liquid extractions of Co(II) and Ni(II) from aqueous solutions containing different concentrations of HCl and NaCl with the aim of obtaining the conditions for best extraction and selectivity in separation. Then, a series of PIMs containing different weight fractions of [P_66614_][Dec] are prepared, characterized, and tested for metal extractions.

## 2. Results and Discussion

### 2.1. Liquid-Liquid Extractions

This section examines how different HCl concentrations impact the extraction of Co(II) and Ni(II) using [P_66614_][Dec]. Such acidic media have been selected as HCl is often used for leaching battery cathodes [55].

**Figure 2:**
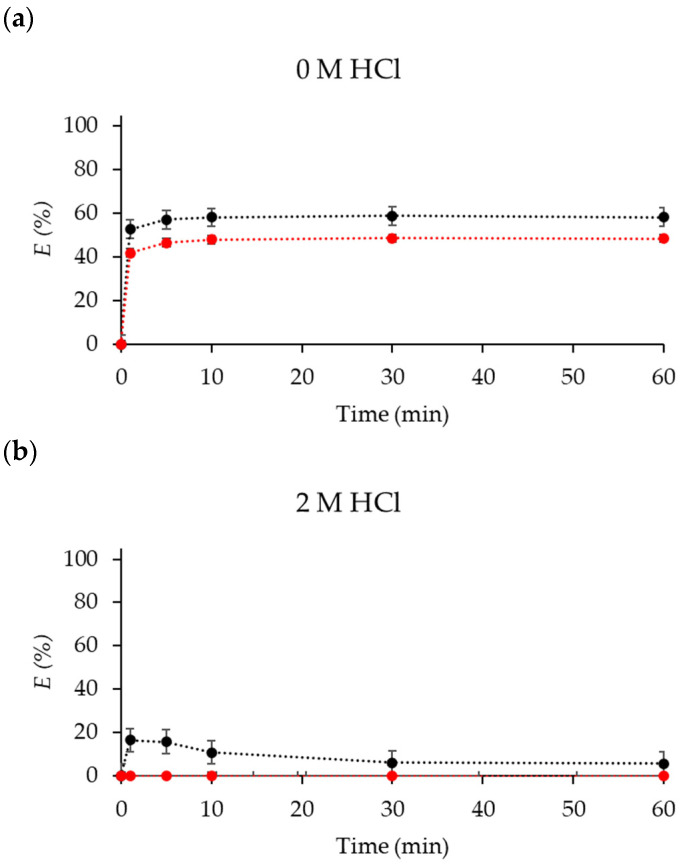

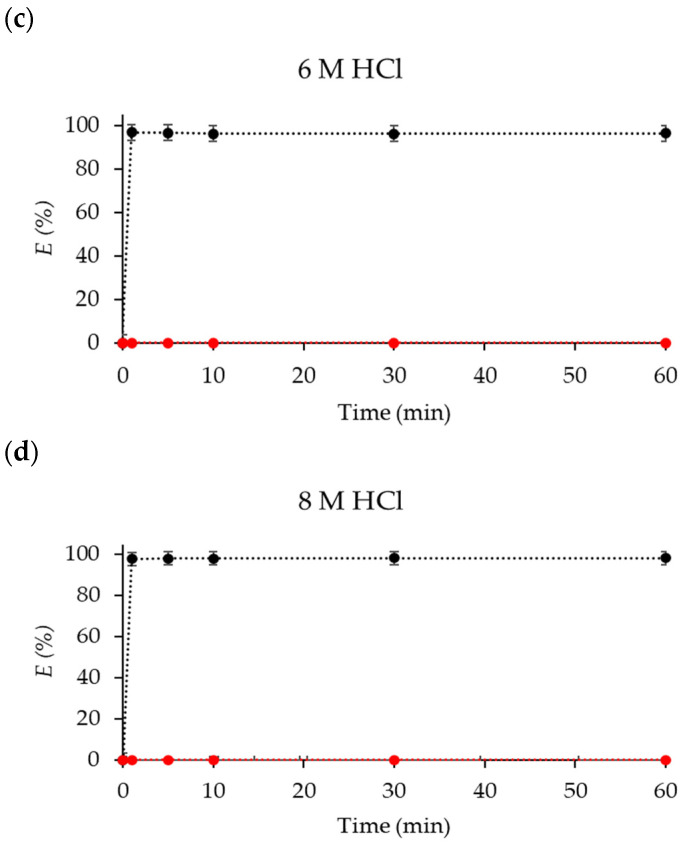
*E*(%) of Co(II) (black) and Ni(II) (red) in (**a**) 0 M HCl, (**b**) 2 M HCl, (**c**) 6 M HCl, and (**d**) 8 M HCl media at different times. Initial concentrations: [Co]_aq_ = [Ni]_aq_ = 50 mM.

Different extraction efficiencies (*E*%) were obtained for Co(II) and Ni(II) as the HCl concentration was increased (Figure 2). The distribution coefficients (*D*) of metal ions between organic and aqueous phase are reported in Table S1.

The remarkable selectivity for Co(II) at 8 M HCl for [P_66614_][Dec] is comparable to the data obtained previously with [P_66614_][Cl] [56,57]. This result was not influenced by the presence of Ni(II), as can be deduced from Figure S3 where the *E*(%) for extractions are from solutions containing Co(II) only.

**Figure 3:**
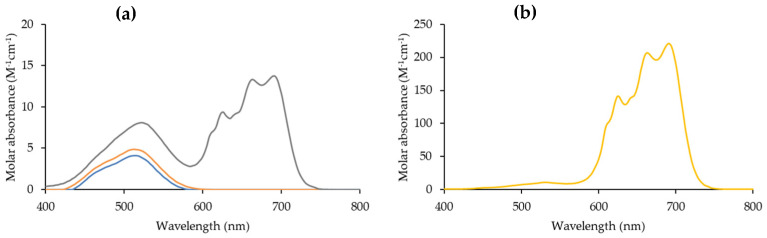
Absorption spectrum of Co(II) aqueous phase in (**a**) 0 M HCl (blue), 2 M HCl (orange), 6 M HCl (grey) and (**b**) 8 M HCl (yellow).

The distinct extraction efficiencies of Co(II) and Ni(II) can be attributed to their different speciation in the aqueous phase [56,58,59,60]. It is well known that in concentrated chloride solutions Co(II) is able to form stable complexes with chloride anions, and different speciation models including up to 1:4 Co:Cl species [61,62,63,64]. Recent studies [65,66] suggest that in the HCl concentration range between 0 M and 11 M the dominant species in solution are the 1:1 [CoCl]^+^ and the tetrahedral 1:4 [CoCl_4_]^2−^ (Figure 3). On the contrary, Ni(II) mainly forms one 1:1 species [67], [NiCl]^+^, which retains the octahedral coordination mode in aqueous solutions (Figure S1).

**Figure 4:**
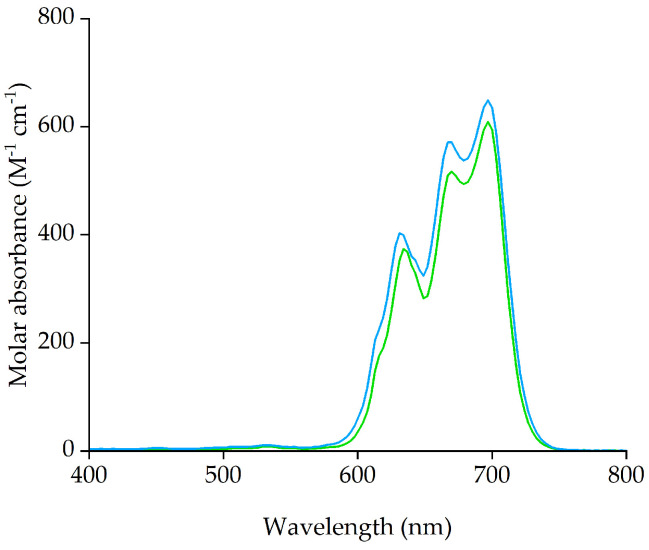
Absorption spectrum of the IL phase after extraction from 8 M HCl in [P_66614_][Dec] (blue) and [P_66614_][Cl] (green).

The UV-Vis absorption spectrum of the IL phase after extraction from 8 M HCl (Figure 4) corresponds to that of the [CoCl_4_]^2−^ complex, as it is nearly superimposable with that recorded after the extraction using [P_66614_][Cl] in the same conditions, where the coordination of tetrahedral Co(II) has been established previously [56].

The extraction from an aqueous solution where CoCl_2_ and NiCl_2_ were dissolved in pure water (measured pH = 5.7) presents an extraction efficiency of 52.7% for Co(II) and 41.9% for Ni(II).

**Figure 5:**
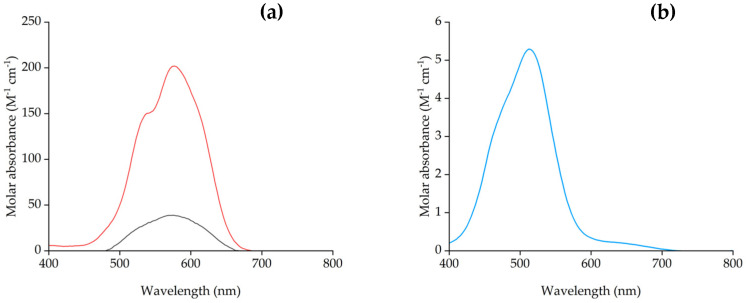
Absorption spectrum of (**a**) CoCl_2_ extracted in [P_66614_][Dec] from aqueous solution (black) λ = 573 nm (ε_573_ = 38.7 M^−1^ cm^−1^), and Co(CH_3_COO)_2_ in dry [P_66614_][Dec] (red) (max. λ = 580 nm, ε_580_ = 201.9 M^−1^ cm^−1^); (**b**) CoCl_2_ in water (blue) (max. λ = 515 nm, ε_515_ = 5.14 M^−1^ cm^−1^).

The spectra for the IL phase Co(II) (Figure 5) with a maximum absorption at λ = 573 nm (ε_573_ = 38.7 M^1^ cm^−1^), is intermediate between that of Co(II) in water (max. λ = 515 nm, ε_515_ = 5.14 M^−1^ cm^−1^) and that of Co(II) acetate salt in anhydrous [P_66614_][Dec] (max. λ = 580 nm, ε_580_ = 201.9 M^−1^ cm^−1^). The spectra of the extracted Ni(II) with [P_66614_][Dec] is depicted in Figure S2 (max. λ = 328 nm, ε_328_ = 15.9 M^−1^ cm^−1^). It can therefore be proposed that in [P_66614_][Dec] both Co(II) and Ni(II) are extracted as octahedral species by coordination with the [Dec]^−^ anions and water in their coordination spheres.

On the basis of the above results, the extraction of Co(II) with [P_66614_][Dec] can be explained using different equilibria depending on the HCl concentration. In absence of chloride (0 M HCl) the extraction occurs through the equilibrium (1) as previously proposed for other phosphonium ILs [68]:

(1) $$M^{2+}+{2Cl}^{-}+\bar{2\left[P_{66614}\right]\left[Dec\right]}⇌\bar{2[P_{66614}]Cl}+\bar{M{Dec}_{2}}$$

M = Co, Ni

As in such conditions the extraction occurs through coordination of the metal ions, the low selectivity towards Co(II) can be explained by the similar affinity of the carboxylate group towards Co(II) and Ni(II).

On the contrary, in the conditions where Co(II) ions exist as anionic chloro-complexes, the following extraction equilibrium [68] can be proposed:

(2) $$[{CoCl}_{4}{]}^{2-}+2H^{+}+\bar{2\left[P_{66614}\right]\left[Dec\right]}⇌\bar{[P_{66614}{]}_{2}\left[{CoCl}_{4}\right]}+\bar{2DecH}$$

Unlike the extractions with [P_66614_][Br] and [P_66614_][Cl], which are based on an anion exchange mechanism [56,57,68,69,70] (i.e., the anion is transferred to the aqueous phase), for [P_66614_][Dec] the protonation/deprotonation state of the IL anion changes [68]. As discussed later (Section 2.3), in the stripping process with pure water, the protons are released to the aqueous phase.

Based on the equilibrium (2), the low extraction from 2 M HCl solution can be explained by the fact that in such condition a negligible amount of [CoCl_4_]^2−^ species is formed (if the model in ref. [65] is assumed), and decanoate anions are protonated due to the high acid concentration, hence the metal ion coordination by [Dec]^−^ anion is suppressed.

**Figure 6:**
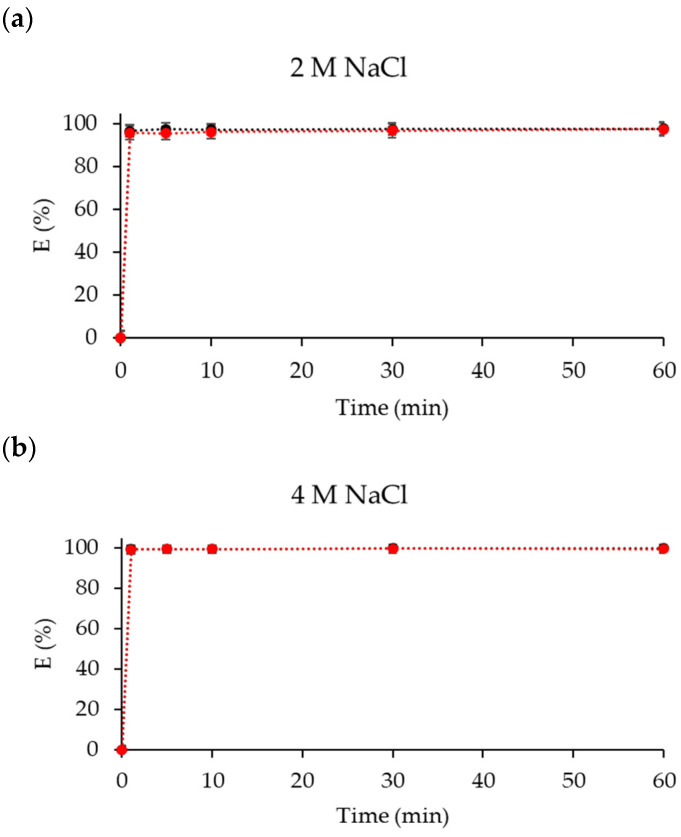
Co(II) (black) and Ni(II) (red) *E(*%) from aqueous NaCl solutions; (**a**) 2 M NaCl, (**b**) 4 M NaCl. [Co]_aq_ = [Ni]_aq_ = 50 mM.

**Figure 7:**
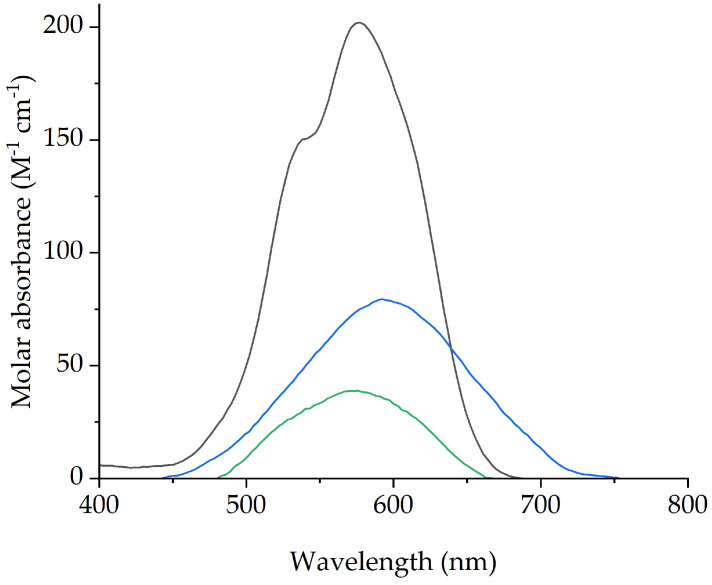
Absorption spectrum of CoCl_2_ extracted in [P_66614_][Dec] from aqueous solution (0 M HCl, green) λ = 573 nm (ε_573_ = 38.7 M^−1^ cm^−1^), Co(CH_3_COO)_2_ in dry [P_66614_][Dec] (black) (max. λ = 580 nm, ε_580_ = 201.9 M^−1^ cm^−1^), and of the [P_66614_][Dec] IL phase after extraction of Co(II) in 2 M NaCl solution (blue) (max. λ = 595 nm, ε_595_ = 79 M^−1^ cm^−1^).

Extraction experiments with NaCl (2 and 4 M) in the feed were also carried out (*D* values in Table S1). At higher concentrations (>5 M) NaCl is not completely soluble. It was found that extraction efficiency of Co(II) using [P_66614_][Dec] increased with increasing chloride concentration, but Ni(II) was also extracted at the same time (Figure 6). The spectra of the IL phase after extraction of Co(II) (Figure 7) suggests a mechanism similar to that from pure water. At higher concentrations of NaCl (˃4 M), a colloidal phase is formed [71].

Interestingly, the spectrum of the IL phase after extraction from 2 M NaCl (Figure 7) indicates that an octahedral Co(II) species is formed, and suggests that the process proceeds through the complexation of the metal ion by decanoate. The fact that the extraction from 2M NaCl is higher than that from water could be assigned to the salting out effect [56].

### 2.2. Effect of Temperature

**Figure 8:**
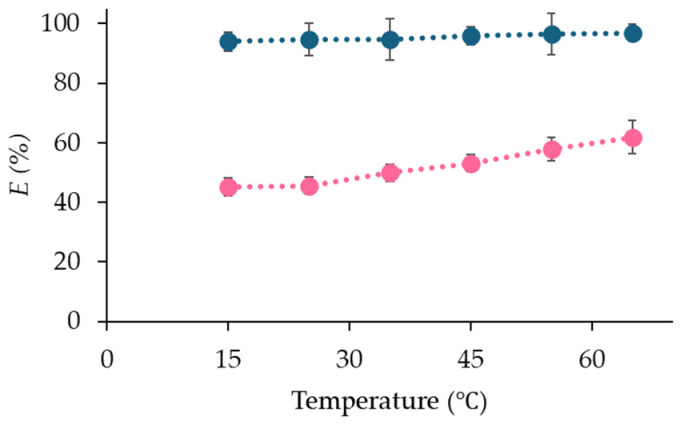
Extraction efficiency of Co(II) from pure water (pink) and from 6 M HCl (blue) at different temperatures.

The extraction of Co(II) was performed at six different temperatures (15–65 °C) from pure water and 6 M HCl (Figure 8).

Temperature does not significantly affect the extraction of Co(II) from HCl. However, when Co(II) is extracted from pure water, the process becomes slightly more favorable at higher temperatures. The fact that *E*(%) is similar at room temperature and elevated temperatures indicates that the separation can be performed without additional heating, resulting in substantial savings in both energy and cost.

### 2.3. Stripping of Co(II)

**Figure 9:**
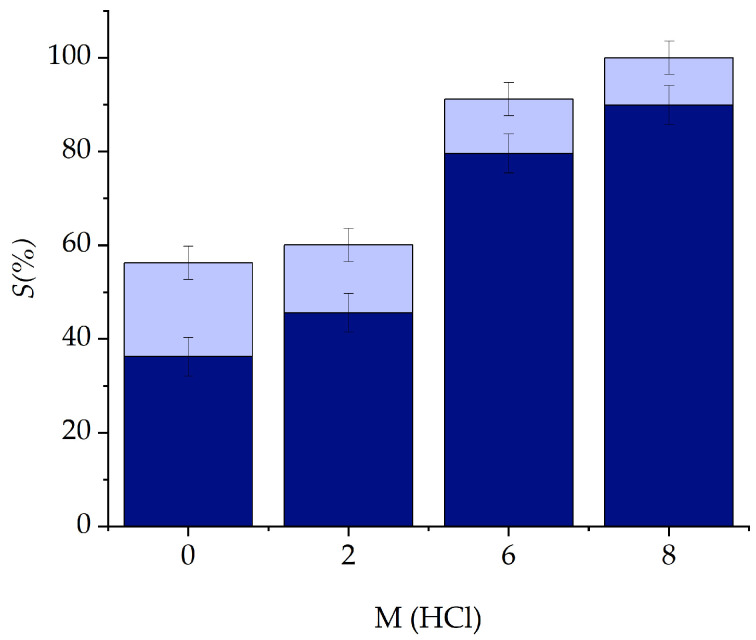
Cumulative stripping (*S*%) of Co(II) from [P_66614_][Dec] extracted from different HCl feeds (M HCl) utilizing only water. Dark blue in one step, dark blue + light blue after two steps.

After the first cycle of equilibration of the metal-containing IL phase with pure water, up to 80% of stripped Co(II) was obtained. More cycles are needed to completely strip the IL of its Co(II) content using only water (Figure 9 and Table S2). The equilibrium (3) is therefore reached:

(3) $$\bar{2\left[P_{66614}\right]\left[Co{Cl}_{4}\right]}+\bar{2DecH}⇌{Co}^{2+}+4{Cl}^{-}+\bar{2\left[P_{66614}\right]\left[Dec\right]}+2H^{+}$$

**Figure 10:**
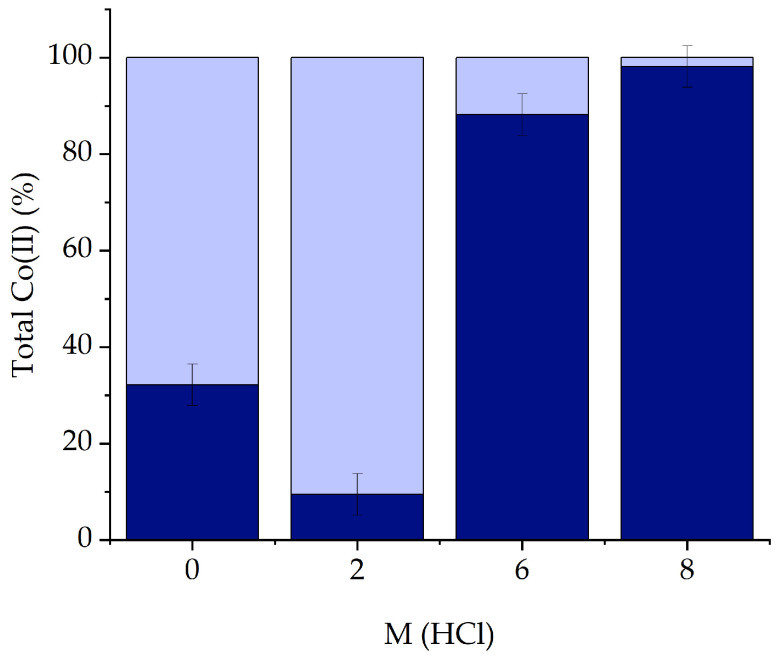
In dark blue, the percentage of recovered Co(II) from 0 to 8 M HCl media. Dark blue + light blue, total Co(II) present in the experiment.

Moreover, the 64.4% of Ni(II) was stripped from the IL phase using water after two consecutive cycles from pure water media. The total Co(II) recovery (Equation (7)) is shown in Figure 10.

The best conditions for separation of Co(II) from Ni(II) are extraction from 8 M HCl and stripping with water, which was implemented in the membrane separation experiment.

### 2.4. Membrane Characterization

PIMs were produced by combining PVC and [P_66614_][Dec] at different weight ratios (20, 50, 70%). Average thickness of the membranes was 0.115 ± 0.02 mm. The resulting PIMs were characterized by means of spectroscopic, mechanical, and thermal properties.

**Figure 11:**
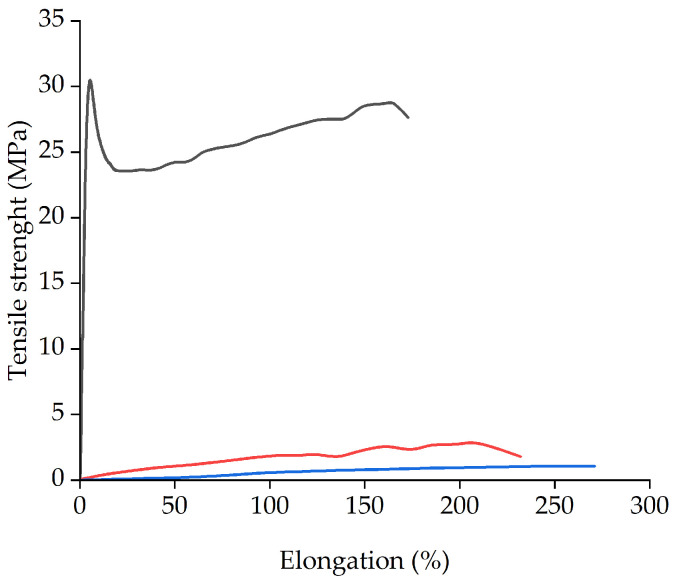
Stress–strain curves of PVC: [P_66614_][Dec] (80:20) black, PVC: [P_66614_][Dec] (50:50) red, and PVC: [P_66614_][Dec] (30:70) blue.

As can be seen in Figure 11, the tensile strength is strongly affected by the composition. The PIM with 20% of [P_66614_][Dec] shows similar behavior as pure PVC [72], while the membranes with 50% and 70% of [P_66614_][Dec] display typical stress–strain curves (Figure 11) for flexible materials [73]. The addition of IL [P_66614_][Dec] to PVC increases elongation at rupture, but decreases the tensile strength of the membrane, which is due to its plasticizing properties [74].

Water-membrane contact angle was measured to assess the effect of the IL on the wettability of the PIM. Good wettability of the membrane is important for successful metal transport [75]. In Table 1 the contact angles are shown for the compositions of the PIMs tested in this work.

The contact angle of the pure PVC (*θ* = 75.7°) (Table 1) is reduced by the addition of [P_66614_][Dec]. Although [P_66614_][Dec] is hydrophobic, its presence can disrupt the regular hydrophobic domains of pure PVC, leading to a modified surface energy that may enhance water affinity to some extent. This increased chain mobility can lead to a smoother surface, which facilitates better water spreading and results in a lower contact angle [76]. A lower contact angle indicates better wettability, which facilitates the initial wetting of the membrane.

**Figure 12:**
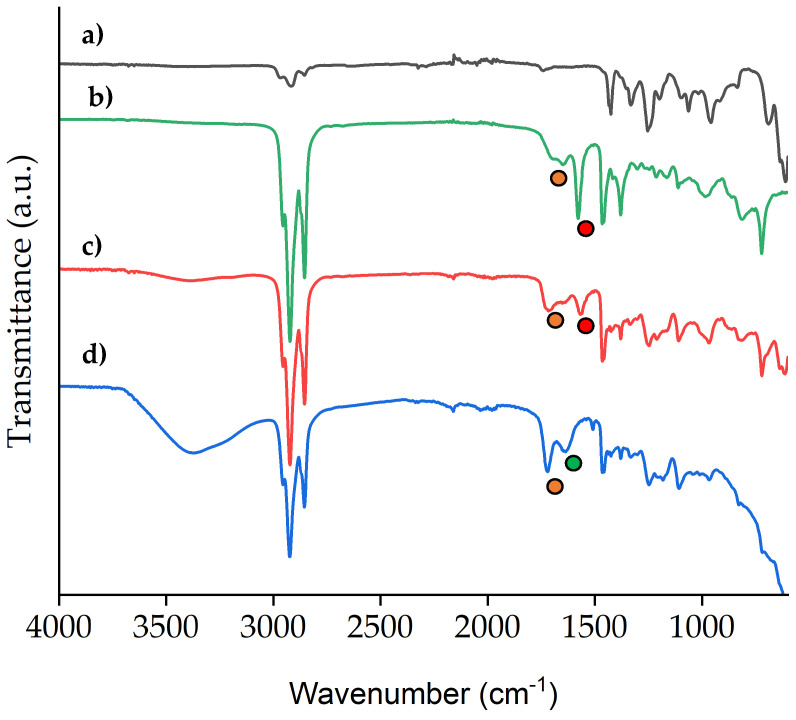
FTIR spectra of (**a**) pure PVC (black); (**b**) pure [P_66614_][Dec] (green); (**c**) PVC: [P_66614_][Dec] (30:70) PIM (red); and (**d**) PVC: [P_66614_][Dec] (30:70) PIM after extraction of Co(II) from 8 M HCl (blue).

The vibrational spectrum of PVC (Figure 12a) is also deeply modified when the IL is incorporated (Figure 12b). For this characterization only the membrane PVC: [P_66614_][Dec] (30:70) was considered, as it was the one with the optimal extraction performance (see Section 2.5).

Firstly, the C-H stretching modes at 2920 cm^−1^ become significantly more intense than in the starting polymer, due to the aliphatic tails of the added [Dec]^−^ anion. New peaks at 1574 and 1728 cm^−1^ (red and orange circles) assigned to the C=O stretching modes of the carboxylate group [77] are present as well. In the used membrane (Figure 12d), the peak at 1574 cm^−1^ disappears, while the peaks at 1640 cm^−1^ (green circle) and 1728 cm^−1^ increase in intensity. This spectral feature shows that the IL is retained in the membrane after use. Moreover, the peaks’ positions in spectrum Figure 12d are diagnostic of the protonation of the carboxylate group [77] which is caused by the prolonged contact with the strongly acidic solution [77]. The latter result is coherent with the proposed equilibrium (2) where the decanoate is protonated when Co(II) is extracted in the IL phase in the PIM. Water is also present in the membrane, as revealed by the broad band centered around 3400 cm^−1^ assigned to the water O-H stretching.

The thermal behavior of PIMs was evaluated by differential scanning calorimetry (DSC) which displays a strong dependence upon composition.

**Figure 13:**
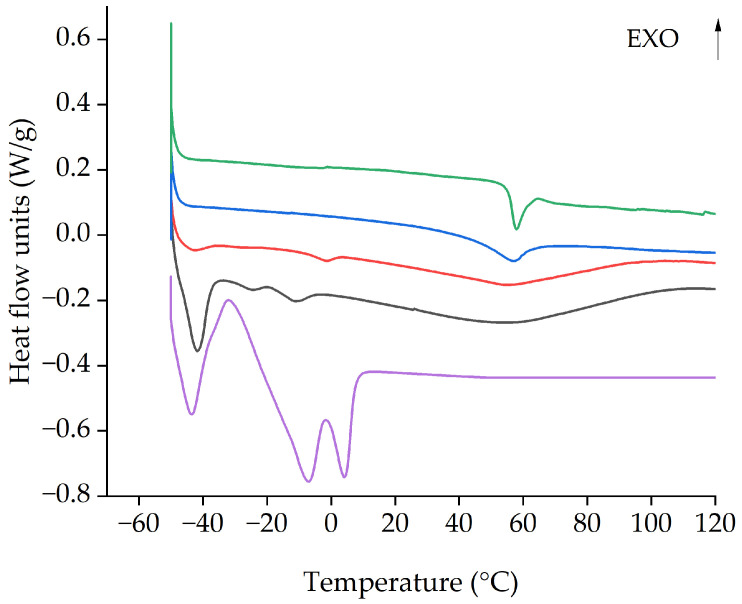
Thermograms of pure PVC (green), PVC: [P_66614_][Dec] (80:20) (blue), PVC: [P_66614_][Dec] (50:50) (red), PVC: [P_66614_][Dec] (30:70) (black), and pure [P_66614_][Dec] (purple).

In Figure 13, the DSC of pure PVC membrane and PVC: [P_66614_][Dec] (80:20) presents the glass transition temperature (T_g_) at 58 °C. The glass transition becomes increasingly broader in the PIMs with an increased fraction of [P_66614_][Dec]. Melting peaks appear at −2 °C in PVC: [P_66614_][Dec] (50:50) (red) and with an increase of the IL fraction shift towards lower temperatures. This feature is clearly related to the included [P_66614_][Dec] as can be seen in the DSC of the pure liquid which shows three melting peaks at −43 °C, −7 °C, and +4 °C.

**Figure 14:**
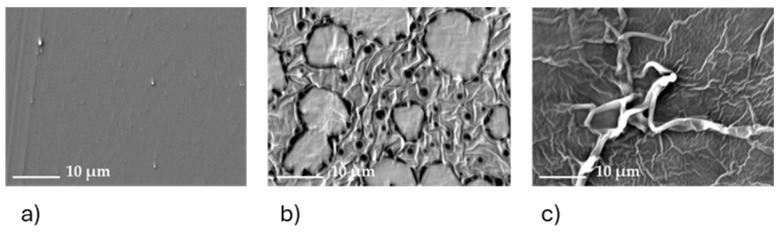
SEM images of (**a**) pure PVC, (**b**) PVC: [P_66614_][Dec] (30:70) before extraction, and (**c**) PVC: [P_66614_][Dec] (30:70) after extraction.

The surface morphology of the starting polymer (Figure 14a) also changes upon inclusion of [P_66614_][Dec] and after use. The initial homogenous surface of PVC is significantly altered by introducing larger granules (around 15 µm) and smaller pore-like structures (around 3 µm) (Figure 14b). These clusters and pores create a rough, heterogeneous surface resulting in a bigger surface area and thus promoting better metal transport. After being in contact with a Co(II)/Ni(II) 8 M HCl solution for 52 h, a roughness in the surface appears (Figure 14c).

### 2.5. Separation of Co(II) from Ni(II) with PIMs

**Figure 15:**
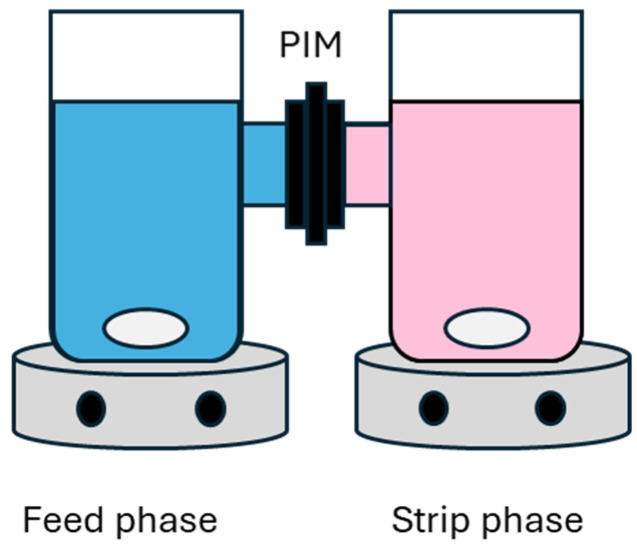
Experimental setup for the separation using the PVC: [P_66614_][Dec] PIM. The volume of each compartment is 50 mL, contact surface with the membrane 4.90 cm^2^, and stirring speed 700 rpm.

Co(II) was separated from Ni(II) utilizing a PIM based on 30% PVC and 70% [P_66614_][Dec] with an experimental setup shown in Figure 15. The membranes with a lower percentage of [P_66614_][Dec] did not display metal extraction within 48 h while the membrane with higher IL content was too fragile to be usable. A possible explanation of this behavior is that the 30:70 composition allows a sufficiently fast diffusion through the membrane to observe extraction in the typical experimental timeframe. The presence of a “threshold” concentration of the carrier in the membrane to observe transport was previously observed for other systems [46,78]. The working conditions were established based on the best performance obtained in the liquid-liquid extraction experiments (feed containing [Co]_aq_ = 10 mM, [Ni]_aq_ = 10 mM in 8 M HCl, pure water in the stripping phase, T = 25 °C).

The permeation of metal through the membrane consists of three steps: (i) absorption of [CoCl_4_]^2−^ into the membrane, (ii) transport through the membrane, and (iii) release of Co(II) from the membrane into the stripping phase (Equation (3)).

**Figure 16:**
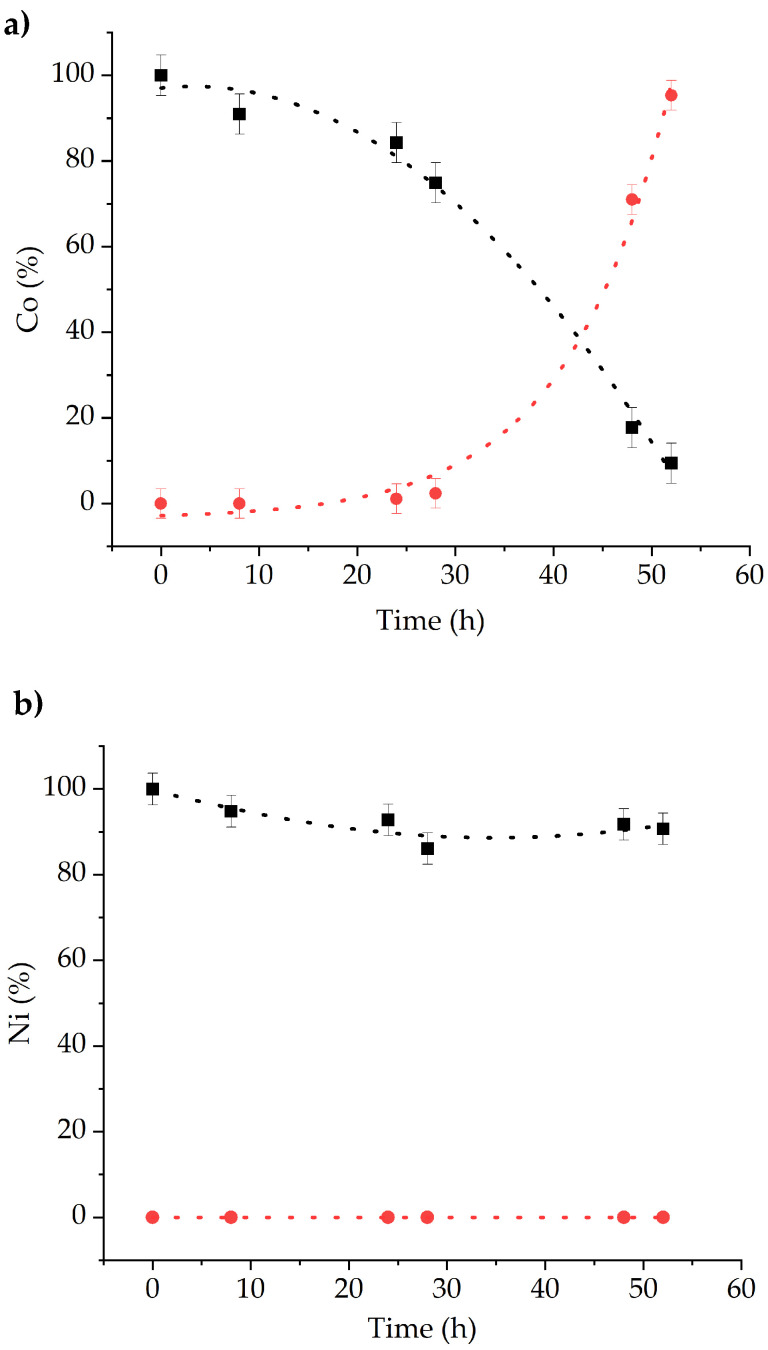
Relative change (%) with respect to initial concentration (10 mM) vs. time of (**a**) Co(II) and (**b**) Ni(II) in the feed (black) and strip (red) phases.

In Figure 16a the relative concentration of Co(II) with respect to the initial one in the feed and stripping phases vs. time is plotted. The concentration in the stripping phase increases slowly until ~30 h when an onset is observed. At 50 h, around the 95% of the initial Co(II) is transferred to the stripping phase, while the metal concentration drops below the detection limit in the feed. This implies that ~5% of Co(II) remains incorporated in the membrane. On the other hand, Ni(II) concentration decreases slightly in the feed (~7%), but it is not detected in the stripping phase (Figure 16b).

The final pH of the stripping phase was measured and found to be <1.0, indicating that protons are transported through the membrane. This is also supported by the FTIR spectrum of the used membrane (Figure 12). The stripping phase also shows a positive reaction (white precipitate formation) upon the addition of AgNO_3_, confirming the transfer of Cl^−^ anions in the aqueous solution.

## 3. Materials and Methods

### 3.1. Chemicals

Trihexyl(tetradecyl)phosphonium decanoate ([P_66614_][Dec]) (>95%) and Trihexyl(tetradecyl)phosphonium chloride ([P_66614_][Cl]) (>95%) was purchased from IoLiTec (Heilbronn, Germany). CoCl_2_·6H_2_O was purchased from JT Baker (Phillipsburg, NJ, USA), NiCl_2_·6H_2_O were ordered from Sigma-Aldrich (Burlington, MA, USA). NaCl was purchased from Honeywell Fluka. HCl (37% solution in water) was ordered from Sigma-Aldrich. PVC (high molecular weight) was purchased from Sigma-Aldrich. THF was purchased from Sigma-Aldrich. All products were used as received, without any further purification.

### 3.2. Extraction and Stripping Experiments

Metal extraction experiments were conducted for several aqueous solutions with different HCl or NaCl concentrations containing CoCl_2_ and NiCl_2_ (total concentration of each metal = 50 mM). For the extractions, 2.0 mL of the aqueous solution and 2.0 mL of the IL phase were stirred at 1500 rpm for variable times ranging from 1 to 60 min at 25 °C. The IL phase was pre-equilibrated with different concentrations of HCl or NaCl for one hour before use.

The temperature was controlled by immersing the sample tube in a thermostatic bath. The experiments were performed from 15 to 65 °C for 5 min in a thermostatic bath.

The total metal content of the water phases was determined using ICP-OES (Agilent 5800, Palo Alto, CA, USA)). Calibration curves were built by analyzing standard solutions in the concentration range for 0–50 mg L^−1^ (Figure S4) and prepared starting from a multi-element standard solution (Merck, Darmstadt, Germany). Argon was used as an internal standard. All the measurements were conducted in triplicate.

The electronic (UV-Vis) spectra of the metal-containing solutions were recorded with a Varian Cary 50 spectrophotometer in a 0.1 and 10 mm quartz cuvette.

The percent extraction (*E*%) is defined as the amount of metal extracted to the IL phase over the total amount of metal in both phases and is given by the following expression (Equation (4)) [39]:

(4) $$E(\%)=\frac{V_{IL}{\left[M\right]}_{IL}}{V_{aq}{\left[M\right]}_{0}}\times 100=\frac{{\left[M\right]}_{0}-{\left[M\right]}_{aq}}{{\left[M\right]}_{0}}\times 100$$

The volumes of the ionic liquid (*V*_IL_) and aqueous phase (*V*_aq_) are the volumes of the organic and aqueous phases, which are equal in our experiments. The molar concentrations [M]_0_, [M]_aq_, and [M]_IL_ are the metal in the initial water phase and in the aqueous and IL phase when the extraction equilibrium is reached.

The *D_M_* (M = Co, Ni) was calculated as follows (Equation (5)) [39]:

(5) $$D_{M}=\frac{{\left[M\right]}_{0}-{\left[M\right]}_{aq}}{{\left[M\right]}_{aq}}$$

Stripping was performed several times, by equilibrating the loaded IL phase with an equal volume of water (2.0 mL) and shaking for 5 min. The aqueous and IL phases were separated for further analysis with ICP-OES. The stripping was evaluated by calculating the *S*(%), using Equation (6) [38]:

(6) $$S\left(\%\right)=\frac{V_{aq,s}{\left[M\right]}_{aq,s}}{V_{IL}\;{\left[M\right]}_{IL}}\times 100=\frac{{\left[M\right]}_{aq,s}}{{\left[M\right]}_{IL}}\times 100$$

where [M]_IL_ is the metal concentration in the IL phase after the extraction, [V]_IL_ is the volume of the IL phase, [V]_aq,s_ is the volume of the aqueous phase used for stripping, and [M]_aq,s_ is the metal concentration in the aqueous phase after stripping. After stripping, the Co(II) recovered (*R*%) was calculated by Equation (7):

(7) $$R\;\left(\%\right)=\frac{{\left[Co\right]}_{extracted\;}}{{\left[Co\right]}_{initial}}\times S(\%)$$

### 3.3. Membrane Preparation

**Figure 17:**
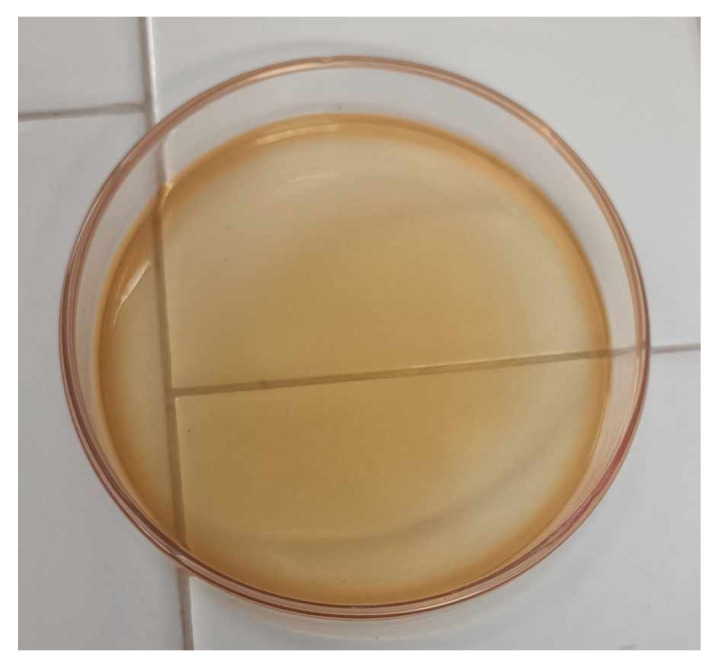
PVC: [P_66614_][Dec] (30:70) membrane after synthesis.

A set of membranes was prepared by dissolving PVC (0.8, 0.5, 0.3, and 0.2 g) and IL [P_66614_][Dec] (0.2, 0.5, 0.7, 0.8 g) in 10 mL of THF. The mixture of PVC and IL was stirred on a magnetic stirrer until dissolution was completed. The total mass of each membrane was ~1 g. After dissolving the PVC and IL, the solution was poured into glass Petri plates (Figure 17) and was left to evaporate overnight [79]. Then, membranes were peeled from the Petri dishes and used without further treatments.

### 3.4. Contact Angle

The surface contact angle of the resultant PVC membranes was measured by a portable video-based goniometer PGX. Deionized water was slowly dropped onto the surface of the specimens. The angle was measured manually by the three-point method. At least five different locations were measured for each specimen. The indoor temperature was 27 ± 0.5 °C.

### 3.5. Elastic Modulus

Samples were manually cut into 10 × 60 mm strips and subjected to tensile test using a 34TM-5 dynamometer (Instron LTD., High Wycombe, UK) equipped with a 5 kN loading cell. Samples were pulled until failure at a 10 mm/min rate. Percentage elongation measures the ability of a material to deform under tensile stress before breaking.

### 3.6. Microscopy

A field-emission-gun scanning electron microscope (FE-SEM Jeol JSM7600F Scanning Electron Microscope, JEOL, Tokyo, Japan) was used to observe and characterize the samples. All images were collected at an acceleration voltage of 15 kV, a distance of 15 mm, and at magnifications ranging between 25× and 10,000×. All samples were sputter-coated (Cressington, Watford, UK) with a thin (2–5 nm) layer of gold to improve their electrical conductivity.

### 3.7. FTIR

ATR spectra of pure PVC and PVC: [P_66614_][Dec] (30:70) membrane before and after extraction were collected using a Fourier transform infrared (FT-IR) spectrometer (Thermo Scientific Nicolet iS-50 FTIR, Monza, Italy) equipped with an ATR module and a deuterated triglycine sulfate (DTGS) detector. Each spectrum was collected using 32 scans and a spectral resolution of 4 cm^−1^. Wavelength varied from 4000 cm^−1^ to 400 cm^−1^.

### 3.8. DSC

Samples were manually cut into approximately 3 × 3 mm squares and weighed to 0.0001 g precision inside 100 µL aluminum pans (Mettler-Toledo, Greifensee, Switzerland). A DSC 3 Stare System differential scanning calorimeter was then used to heat the samples from −50 °C to 120 °C at a 10 °C/min heating rate under continuous nitrogen flow (20 mL/min). Glass transition temperature and peak enthalpies were obtained by elaborating themograms using the STARe software (ver. 16.10, Mettler-Toledo).

### 3.9. Extraction with PIMs

Extractions using PIMs were performed in a setup depicted in Figure 15. The setup was purchased from Tecnovetro s.r.l (Monza, Italy). The volume of the feed and stripping phases were 50 mL each, at room temperature, with a stirring speed of 700 rpm and a membrane contact surface of 4.90 cm^2^.

To test the performance of the PVC: [P_66614_][Dec] (30:70) membrane for the separation of Co(II) from Ni(II), an 8 M HCl solution containing CoCl_2_ and NiCl_2_ (metal concentration 10 mM) was prepared and employed as the feed phase. Pure water was used as the stripping phase. The extractions were conducted at room temperature. Feed and stripping phases were periodically sampled, and the metal content was analyzed using ICP-OES.

### 3.10. Extraction Efficiency in Membrane Separation

The change of the metal concentration in the feed phase was calculated by Equation (8), while the change of the metal concentration in the stripping phase was calculated by Equation (9):

(8) $$\%\;M_{feed}=100-\left(\frac{\left[M_{start}\right]-\left[M_{t,\;feed}\right]}{\left[M_{start}\right]}\right)\times 100\%$$

(9) $$\%\;M_{strip}=100-\left(\frac{\left[M_{start}\right]-\left[M_{t,\;strip}\right]}{\left[M_{start}\right]}\right)\times 100\%$$

where [M_start_] is the initial metal concentration in the feed, [M_t,feed_] is a concentration of metal in the feed phase after t amount of time, and [M_t,strip_] is a concentration of metal in the stripping phase after certain (t) amount of time. 

## 4. Conclusions

This work shows the applicability of a carboxylic acid containing IL in the Co/Ni separation both as in liquid-liquid extraction and supported on a polymeric membrane.

The efficiency obtained of the extraction of Ni(II) and Co(II) from concentrated HCl solutions shows distinct performance as chloride ion concentrations vary, which is explained by the formation of different speciation of these two metal ions. When extraction occurs from water and NaCl solutions, Co(II) and Ni(II) are both extracted through the coordination of decanoate anions, and a low selectivity for Co(II) is obtained. When extractions are performed from concentrated HCl solutions, the decanoate anion is protonated and is not able to bind metal ions. In such conditions Co(II) is selectively extracted as tetrachloride species. Notably, the best performance in terms of *E*(%) and selectivity towards Co(II) is obtained when the feed is above 6 M HCl. Stripping was performed by using deionized water, which allowed a recovery close to the 100% of the total Co(II) in the feed solution.

PIMs formulated with PVC and the IL [P_66614_][Dec] successfully extracted Co(II), when a PVC: Dec ratio of 30:70 was employed. The high selectivity for Co(II) over Ni(II) was comparable to that obtained from liquid-liquid extraction. The use of PIMs reduces the amount of IL required and allows the recovery of Co(II) in a single step. However, the extractions with PIMs in this work require a significantly longer time with respect to liquid-liquid experiments. Even so, the high selectivity obtained with the PVC: Dec (30:70) PIM combined with durability in acidic conditions constitute a starting point for further developments towards practical applications. Several improvements of different aspects of IL-based PIMs have been discussed in a recent review [80]. Another limitation for a scale-up is the high concentration of HCl acid required to achieve a high selectivity, which poses environmental and safety concerns. In this context, the improvement of the process kinetics and the use of less aggressive acids would give a great benefit.

## Figures and Tables

**Table 1:** Contact angle dependence on membrane % weight composition.

PVC (%)	[P_66614_][Dec] (%)	Contact Angle (*θ*)
100	0	75.7 ± 0.5
80	20	74.4 ± 1.6
50	50	51.0 ± 2.8
30	70	28.1 ± 5.4

## Data Availability

Data are available within the manuscript and Supplementary Materials. Further inquiries can be directed to the authors.

## Supplementary Materials

The following supporting information can be downloaded at: https://www.mdpi.com/article/10.3390/molecules29194545/s1, Figure S1. Absorption spectrum of Ni(II) aqueous phase in 0 M HCl (blue), 2 M HCl (orange), 6 M HCl (grey), and 8 M HCl (yellow). Figure S2. Ni(II) in [P_66614_][Dec] IL phase after extraction from 0 M HCl. Figure S3. *E*(%) of Co(II) in [P_66614_][Dec] containing variable concentrations of HCl (a) and NaCl (b) [Co]_aq_ = 50 mM. Figure S4. ICP-OES calibration curves for (a) Co(II), (b) Ni(II). Table S1. *D* of Co(II) and Ni(II) after extraction with [P_66614_][Dec] from HCl and NaCl media. Table S2. Cumulative stripping (S%) of Co(II) from [P_66614_][Dec] extracted from different HCl feeds (M HCl) utilizing only water.
