# Supplementary material for: Selective Co(II) and Ni(II) Separation Using the Trihexyl(tetradecyl)phosphonium Decanoate Ionic Liquid
Source: Molecules. 2024 Sep 25;29(19):4545. doi: 10.3390/molecules29194545 (PMC11477661; doi:10.3390/molecules29194545)
Supplement: Supplementary file 1 [file molecules-29-04545-s001.zip › molecules-3196477-supplementary.pdf]

molecules

Selective Co(II) and Ni(II) Separation using the Tri-hexyl(tetradecyl)phosphonium Decanoate Ionic Liquid

Anđela Kovačević [1], José Alejandro Ricardo García [1], Marilena Tolazzi [1], Andrea Melchior [1,*] and Martina Sanadar [2]

[1] Polytechnic Department of Engineering, University of Udine, Chemical Technologies Laboratories, via Cotonificio 108, 33100, Udine, Italy; kovacevic.andela@spes.uniud.it (A.K.); ricardogarcia.josealejandro@spes.uniud.it (J.A.R.G.); marilena.tolazzi@uniud.it (M.T.)
[2] Centre de Biophysique Moléculaire CNRS UPR 4301, Université d'Orléans, Rue Charles Sadron, 45071 Orléans Cedex 2, France; martina.sanadar@cnrs-orleans.fr
* Correspondence: andrea.melchior@uniud.it

Supplementary Information

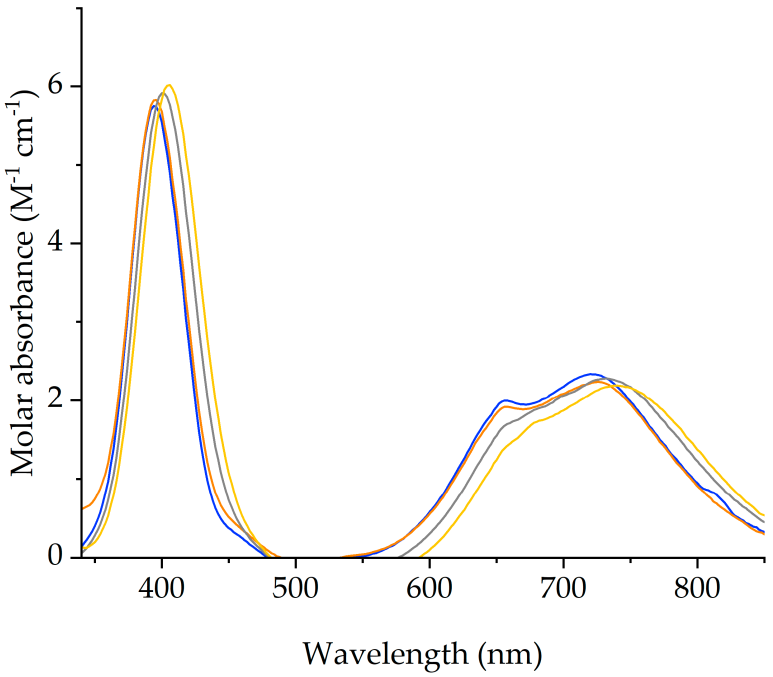

Figure S1. Absorption spectrum of Ni(II) aqueous phase in 0 M HCl (blue), 2 M HCl (orange), 6 M HCl (grey) and 8 M HCl (yellow).

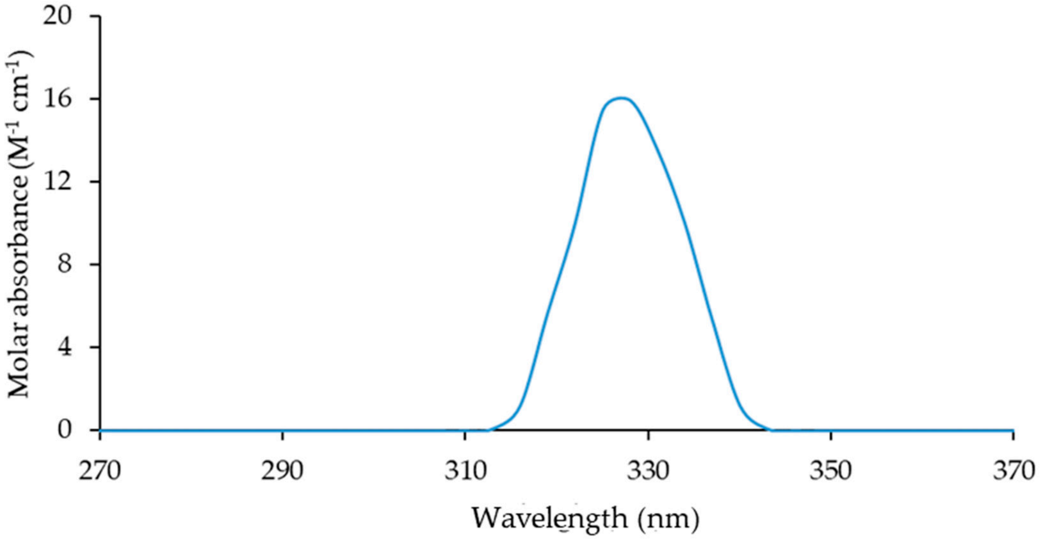

Figure S2. Ni(II) in [P_{66614}][Dec] IL phase after extraction from 0 M HCl.

(a)

(b)

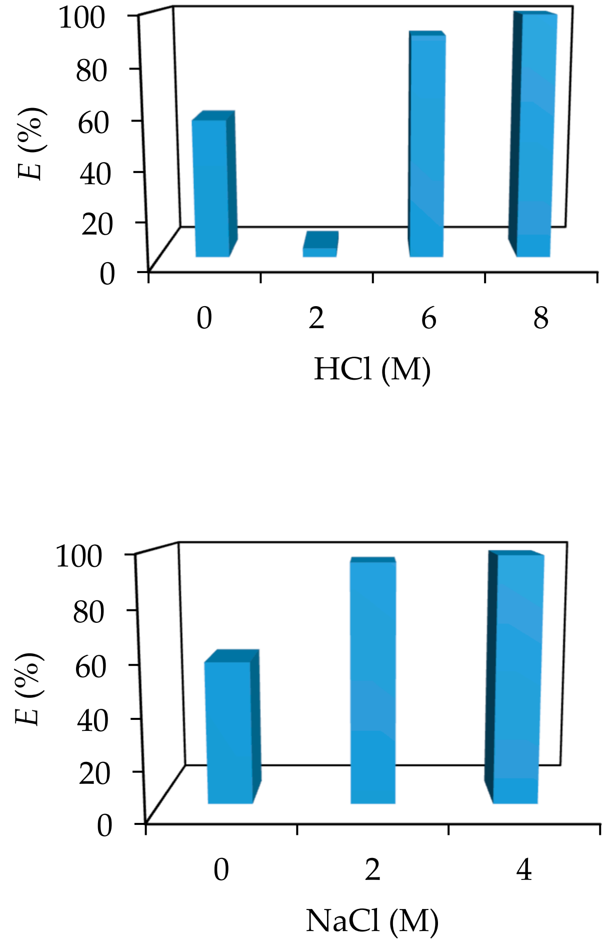

Figure S3. *E*(%) of Co(II) in [P_{66614}][Dec] from the aqueous solutions containing variable concentrations of HCl (a) and NaCl (b). $[Co]_{aq}$= 50 mM.

a) b)

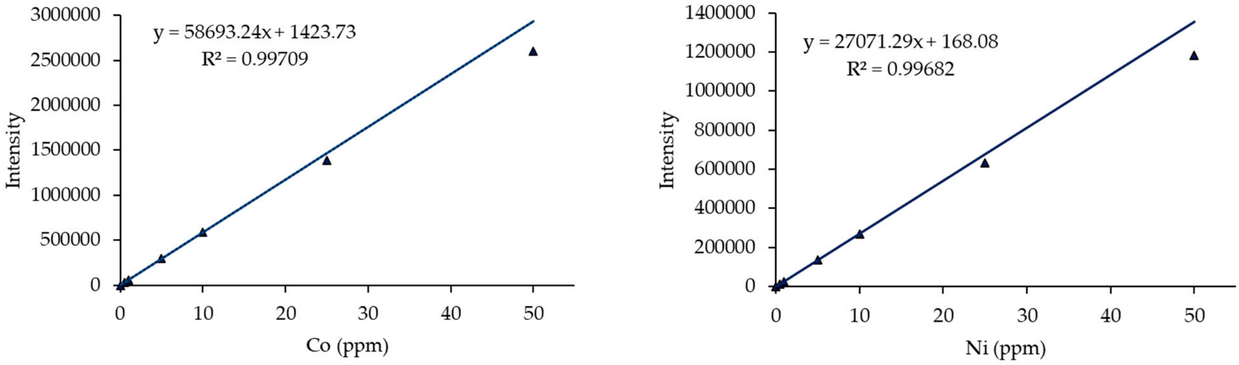

Figure S4. ICP-OES calibration curves for (a) Co(II), (b) Ni(II).

Table S1. *D* of Co(II) and Ni(II) after extraction with [P_{66614}][Dec] from HCl and NaCl media.

	**HCl**		**NaCl**	
	Co(II)	Ni(II)	Co(II)	Ni(II)
0 M	13.3	8.7	13.3	8.7
2 M	1.9	0.0	43.3	23.4
4 M	-	-	264.9	195.1
6 M	299.2	0.0	-	-
8 M	531.7	0.0	-	-

Table S2. Cumulative stripping (*S* %) of Co(II) from [P_{66614}][Dec] extracted from different HCl feeds (M HCl) utilizing only water.

**S (%)**	**1st step**	**2nd step**
0 M HCl	36.2	56.3
2 M HCl	45.6	60.1
6 M HCl	79.6	91.1
8 M HCl	89.9	100.0
